# Cellulose Nanocrystals as Template for Improving the Crystallinity of Two-Dimensional Covalent Organic Framework Films

**DOI:** 10.3390/polym13101561

**Published:** 2021-05-13

**Authors:** Yue Li, Zhaowei Ou, Baokun Liang, Jing Yang, Ruilian Chen, Haoyuan Qi, Ute Kaiser, Wei Hong, Xudong Chen, Liangwei Du, Wei Liu, Zhikun Zheng

**Affiliations:** 1 College of Chemistry and Chemical Engineering, Guangxi University, Nanning 530004, China; LeeYe8@126.com; 2 Key Laboratory for Polymeric Composite and Functional Materials of Ministry of Education, School of Chemistry, and State Key Laboratory of Optoelectronic Materials and Technologies, Sun Yat-Sen University, Guangzhou 510275, China; ouzhw6@126.com (Z.O.); chenrlian@mail2.sysu.edu.cn (R.C.); hongwei9@mail.sysu.edu.cn (W.H.); cescxd@mail.sysu.edu.cn (X.C.); 3 Electron Microscopy Group of Materials Science, Central Facility of Electron Microscopy, Universität Ulm, 89081 Ulm, Germany; baokun.liang@uni-ulm.de (B.L.); haoyuan.qi@uni-ulm.de (H.Q.); ute.kaiser@uni-ulm.de (U.K.); 4 Key Laboratory of Low-Carbon Chemistry & Energy Conservation of Guangdong Province, Key Laboratory for Polymeric Composite and Functional Materials of Ministry of Education, School of Materials Science and Engineering, Sun Yat-Sen University, Guangzhou 510275, China; yangj329@mail.sysu.edu.cn; 5 Center for Advancing Electronics Dresden (CFAED) & Faculty of Chemistry and Food Chemistry, Technische Universität Dresden, 01062 Dresden, Germany

**Keywords:** cellulose nanocrystals, covalent organic framework, crystallinity, film

## Abstract

Despite the rapid development of two-dimensional covalent organic frameworks (2D COFs) in recent years, it remains a great challenge to synthesize highly crystalline COF materials. Here, a CNC-assisted approach was adopted to synthesize high crystallinity COF materials. A series of 2D COF films were synthesized at the air–water interface by using cellulose nanocrystals (CNCs) as the template. The occurrence of Schiff reactions based on the imine bond was demonstrated by Raman spectroscopy and Fourier transform infrared spectroscopy (FTIR). Scanning electron microscopy (SEM) exhibited the appearances of 2D COF films were flower-like. When CNCs were added to a certain extent, the size of a single petal in the flowers gradually increased with the amount of CNCs. The film with large petals was characterized by Ultraviolet–Visible diffuse reflectance spectroscopy (UV–Vis DRS), X-ray photoelectron spectroscopy (XPS), transmission electron microscopy (TEM), and selected area electron diffraction (SAED). In UV–Vis DRS curves, the S-band of COF-366 film was red-shifted by 24 nm compared with that of 5,10,15,20-tetrakis(4-aminophenyl)-21H,23H-porphyrin (TAPP), confirming the existence of extended conjugation in COF-366 film. XPS was used to identify the surface composition of the sample. The N1s signal of the film indicated that each TAPP formed four imine bonds with 2,5-dihydroxyterephthalaldehyde (DHTA) in COF-366 film. TEM images showed that CNCs had an influence on the crystal size. It was observed from SAED that the crystallinity of the film with CNCs was higher than the film without CNCs. This work provided a new template for improving the crystallinity of 2D COF films.

## 1. Introduction

Two-dimensional covalent organic frameworks (2D COFs), as a new class of crystalline porous materials [1,2,3], have attracted great attention and exhibited broad application prospects in many fields such as gas adsorption and separation [4,5], catalysis [6,7,8], electrochemistry [9,10], molecular electronics [11], and so on. Imine-based COFs have a wide range of application prospects because they showed excellent stability for kinds of environments. For example, they can still maintain a good configuration in organic solvents and will not decompose at high temperatures.

The common COF materials are COF powder and COF film. Although COF powder has a nanoscale pore size, the application is limited due to its insolubility in common solvents and difficulty in processing. For further practical applications, new strategies are needed to prepare COFs into ordered films. 2D COF films with precisely defined pore size, good chemical stability, and rigid structure are considered as excellent candidate materials for separation films [12]. Interfacial polymerization can directly polymerize monomers to form films through a bottom-up method, which has been widely used to prepare 2D COF films at the interface of two phases [13,14,15].

Until now, the synthesis of highly crystalline COF films remains difficult. Typically, COF is synthesized through dynamic reversible covalent bonds. The reason is that reversible chemical bonds have self-repair and error correction functions during the crystallization process. In the process of reversible covalent bond formation and extension, if any bond formation occurs in an undesired direction, the system can repair it through reverse reaction and chemical bond recombination [16]. However, due to the poorly controlled nucleation and growth processes, the resulting 2D COFs usually form insoluble amorphous or polycrystalline thin films with small crystalline domains (usually less than 50 nm) [17,18].

To meet these challenges, many approaches have been attempted. Burke and co-workers [19] demonstrated that acid-mediated exfoliation reactivated the imine bond exchange, which caused the COF particles to partially decompose and subsequently repolymerize during film formation, and the resulting film was highly crystalline. Sahabudeen and co-workers [20] synthesized highly crystalline films with the assistance of small molecular surfactants. In addition, template-assisted interfacial synthesis of COF films has been proven to be a feasible method to improve film crystallinity [21].

Cellulose is a macromolecular polysaccharide composed of glucose, which is mainly distributed in some higher plants, and several marine animals. Cellulose nanocrystals (CNCs) can be easily obtained from cellulose by sulfuric acid hydrolysis [22,23,24]. As the amorphous regions in the cellulose are removed by acid hydrolysis, the resulting CNCs often showed a high crystallinity and a high aspect ratio. These characteristics of CNCs make it a feasible template for the preparation of crystalline material. Many crystalline materials including silver nanoparticles (AgNPs) [25], poly(L-lactide) (PLLA) [26,27], and metal organic frameworks (MOFs) [28] have been synthesized via using CNCs as template. As far as we know, the combination of CNCs and COFs has not yet been explored.

Herein, we synthesized imine-based 2D COF films at the air–water interface assisted by CNCs. With CNCs as the template and p-toluenesulfonic acid monohydrate (PTSA) as the catalyst, two monomers of 5,10,15,20-tetrakis(4-aminophenyl)-21H,23H-porphyrin (TAPP) and 2,5-dihydroxyterephthalaldehyde (DHTA) slowly underwent two-dimensional polymerization at the interface between air and water to form the 2D COF films with flower-like morphology. The size of a single petal in the flowers varied to a certain extent with different CNC amounts. In addition, it was observed that the film with large petals has high crystallinity with square lattice. This work provided a new template for improving the crystallinity of 2D COF films, which broadens the potential application of CNCs.

## 2. Materials and Methods

### 2.1. Materials

TAPP (purity 98%) and DHTA (purity 98%) were obtained from Jilin Extension Technology Co. Ltd. (Jilin, China). PTSA (purity 98%) was obtained from Shanghai Aladdin Biochemical Technology Co. Ltd. (Shanghai, China). Tetrahydrofuran (THF, purity 99.7%) was obtained from Guangzhou Lige Technology Co. Ltd. (Guangzhou, China). Water was purified with a Milli-Q system.

### 2.2. Preparation of CNCs

CNCs were synthesized by a well-known sulfuric acid hydrolysis method with few modifications [29,30,31]. Commercial bagasse pulp was hydrolyzed in 64 wt% sulfuric acid (0.1 g of pulp per milliliter sulfuric acid solution) at 50 °C with vigorous stirring. After 60 min, hydrolysis was stopped with cold water (about 10 times the volume of the acid solution used) and the reaction solution remained static at room temperature overnight to complete precipitation. The supernatant was poured out, and the precipitate in the lower layer was collected and washed three times with water at 4 °C to remove the soluble cellulose materials. To remove the excess sulfuric acid, the CNC suspension was placed in dialysis bag of 12,000–14,000 molecular weight cut-off and dialyzed in water for 60 h. Finally, the suspension in the dialysis bag was collected and condensed to the desired concentration (3%) by the far-infrared dryer.

### 2.3. Synthesis of 2D COF Films

TAPP (20 mg, 0.03 mmol) was dissolved in 20 mL PTSA aqueous solution (2 mol/L). DHTA (20 mg, 0.12 mmol), and PTSA (1.922 g, 10 mmol) were dissolved in 250 mL H_2_O. The solutions were sonicated for at least 30 min.

The 0, 10, 20, 60, and 100 μL CNC suspensions were spread into 26 mL water, respectively, and remained static for 30 min to make the CNCs fully diffused in this aqueous solution. Then, 200 μL TAPP solution was injected into the water phase from the bottom of the solution. Injecting from above caused a large amount of TAPP to exist in the form of aggregation at the air–water interface, and the fragmented film was obtained after the reaction was completed. After kept undisturbed for 1 h, 3.7 mL DHTA was injected moderately from the bottom of the solution to minimize the disturbance on CNCs and TAPP at the air–water interface, and allowed DHTA to diffuse slowly from the bottom to replace the sulfonate groups bound to the amine group on TAPP, which was conducive to the formation of crystals. The reaction solution was maintained statically at room temperature. After seven days, the COF films were obtained.

**Figure 1:**
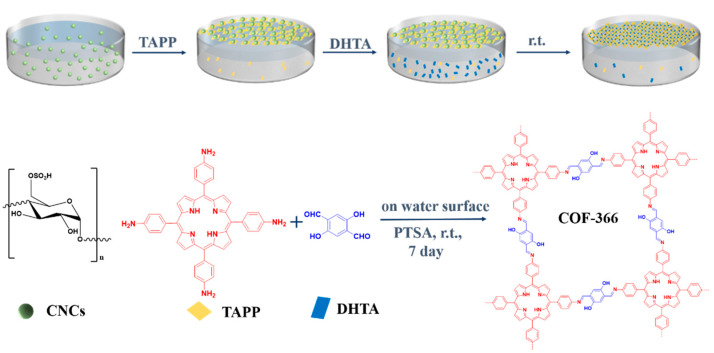
Synthesis process and reaction scheme of the COF-366 film at the air–water interface by using CNCs as a template.

Figure 1 illustrated the synthesis process and reaction scheme of COF-366 film at the air–water interface. CNCs were first added and rested for a period to make it fully diffused, and the molecular weight of CNCs was between 10,000–30,000. Then, TAPP was added and the pH of the system was approximately 2.5. The sulfate groups (–OSO_3_H) on the CNCs were deprotonated under this pH, which could interact with the protonated amine group (–NH_3_^+^) of TAPP to form CNC–surfactants at the interface [32]. CNC–surfactants with high surface activity assembled at the air–water interface could guide the preorganization of the monomer and promote the 2D polymerization. Afterward, DHTA was added into the aqueous phase and spread to the interface. Meanwhile, the imine bonds between amine (–NH_3_^+^) of TAPP and aldehyde groups (–CHO–) of DHTA gradually generated. The reaction solution was kept for seven days at room temperature, and finally, the films formed at the interface.

In the presence of CNCs and PTSA, the monomers of TAPP and DHTA underwent two-dimensional polymerization at the air–water interface to form COF-366 film (Figure S1a). In the absence of CNCs, the film can also be generated at the interface (Figure S1b), however, without PTSA, the COF-366 film cannot be formed at the interface between air and water (Figure S1c). Therefore, PTSA played an important role in film formation. In this reaction system, TAPP and DHTA constituted the basic framework structure, the co-reagent PTSA was used as the acid catalyst to help the reaction proceed [33], and CNCs were used as a template to improve the crystallinity of the film.

### 2.4. Instruments and Measurements

**Figure 2:**
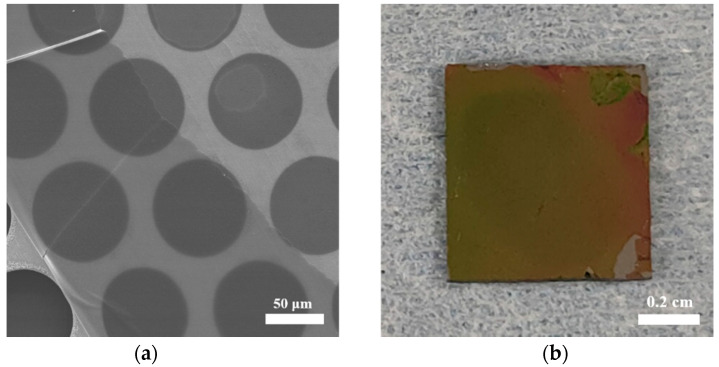
(**a**) SEM image of the COF-366 film on carbon-coated copper grids; (**b**) Optical image of the film on silicon wafer.

The film can be easily transferred to any substrate such as carbon-coated copper grids, silicon wafer, quartz plate, gold-coated silicon wafer and copper grids, which were helpful for the following characterization. It is noted that the transfer process and the type of substrate did not affect the film structure. A few cracks, which may be caused by the drying or transfer process, have been observed in the SEM image (Figure 2a). Raman spectroscopy was performed after the film was transferred to the silicon wafer (Figure 2b).

Raman spectra of the 2D COF films were performed on a confocal Raman spectrometer (inVia Qontor, Renishaw, London, UK) and excited by a 532 nm laser. Raman spectra of DHTA and TAPP were recorded on a FT-Raman spectrometer (Nicolet NXR 9650, Thermo Fisher Scientific, Waltham, MA, USA). The films were horizontally transferred to the silicon wafers, rinsed with THF, and then dried in the air. Monomers and CNCs were spread directly on the silicon wafers in powder and film forms.

SEM images of the films on carbon-coated copper grids were obtained by using a Hitachi S-4800 field emission SEM (JEOL, Tokyo, Japan) at the accelerating voltage of 10.0 kV.

Atomic force microscopy (AFM) was performed on a Dimension FastScan (Bruker, Karlsruhe, Germany) in peak force tapping mode, and the 2D COF films were transferred to the silicon wafers.

The UV–Vis spectra of the 2D COF film with 100 μL CNCs and TAPP were recorded on a UV–Vis–NIR Spectrophotometer (PerkinElmer Lambda 950, PerkinElmer Co., Waltham, MA, USA). The film was horizontally transferred to the silicon wafer, and washed with THF, then air-dried. TAPP was dissolved in THF solvent and sonicated for 5 min. Aliquots of the solution were dropped on the quartz plate until the quartz plate was fully covered by the solution, then dried at room temperature overnight.

FTIR measurements of the 2D COF film with 100 μL CNCs, TAPP, and DHTA were carried out on an attenuated total reflectance-Fourier transform infrared (ATR-FTIR) spectrometer (Perkin Elmer Co., Waltham, MA, USA). The film was horizontally transferred on the surface of the copper sheet. In order to obtain appreciable signal intensity, this transfer was repeated eight times. Monomers were spread directly on the copper sheet in powder form.

XPS spectrum of the 2D COF film with 100 μL CNCs was recorded on Nexsa electron energy spectrometer (Thermo Fisher Scientific, Waltham, MA, USA). The film was transferred to a gold-coated silicon wafer, and rinsed with THF, and then dried in air.

TEM and SAED images of the films with 0, 100 μL CNCs transferred to copper grids were obtained by FEI Titan 80-300 microscopy (FEI, Hillsboro, OR, USA) operated with the acceleration voltage of 300 kV.

## 3. Results and Discussion

### 3.1. Raman Spectra and FTIR Spectra of the Films with Different CNC Amounts

**Figure 3:**
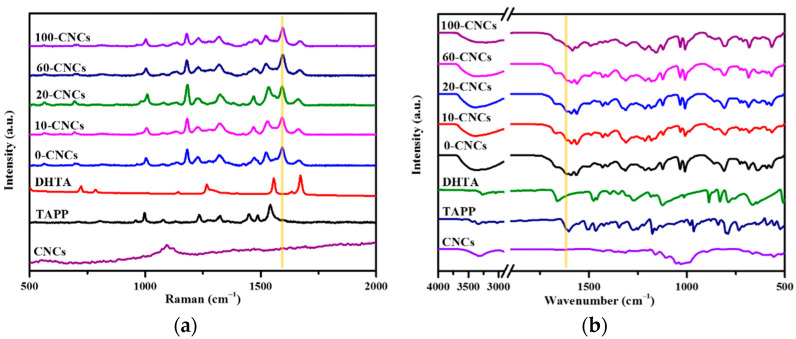
(**a**) Raman spectra of the films with 0, 10, 20, 60, 100 μL CNCs and monomers; (**b**) FTIR spectra of the films with 0, 10, 20, 60, 100 μL CNCs and monomers.

A series of films were characterized to confirm whether there was –C=N-generation associated with the COF formation by Raman spectroscopy and FTIR spectroscopy (Figure 3). In the Raman spectra (Figure 3a), TAPP clearly showed broad bands at 998 cm^−1^, 1282 cm^−1^, and 1327 cm^−1^, which corresponded to v(pyrrole breath), v(phenyl-NH_2_), and v(pyrrole quarter-ring), respectively. DHTA showed an obvious vibration of the C=O group at 1672 cm^−1^. The COF-366 films with different amounts of CNCs showed similar Raman spectra. In the films, the characteristic peaks at 1282 cm^−1^ and 1672 cm^−1^ of the two monomers disappeared, meanwhile, the C=N bond stretching vibration at 1593 cm^−1^ was generated [34,35]. The Raman spectra demonstrated the formation of imine bonds in the Schiff reaction.

The similar phenomenon can be observed in FTIR spectra as the complementary characterization of Raman spectroscopy (Figure 3b). The wavenumber of COF-366 films appeared at 1616 cm^−1^, which corresponded to the stretching vibration of –C=N-bond, meanwhile, the N-H stretching band at 3341 cm^−1^ for TAPP [36] and the aldehyde C=O stretch at 1663 cm^−1^ for DHTA [37] disappeared in the spectra of the COF-366 films. These results of the Raman spectra and FTIR spectra demonstrated the occurrence of the Schiff reaction based on imine bonds.

### 3.2. The Influence of CNC Amount on Morphology

**Figure 4:**
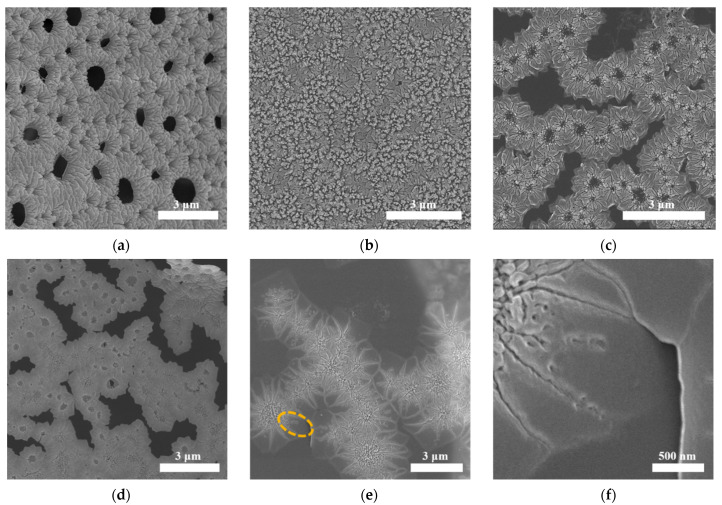
(**a**–**e**) SEM images of COF-366 film with 0, 10, 20, 60, 100 μL CNCs, respectively; (**f**) Detailed SEM image of COF-366 film with 100 μL CNCs.

To understand the influence of different amounts of CNCs on COF-366 films, a series of COF films with 0, 10, 20, 60, 100 μL CNCs were prepared. SEM was used to observe the influence of the amount of CNCs on film morphology. The flower-like morphology of these COF films can be clearly seen in Figure 4a–e. Without CNCs, the morphology of the flower was mainly composed of petals and the average size of a single petal in this film was about 0.3 μm (Figure 4a). AFM presented the petal surface with a roughness of 15.6 ± 1.3 nm (Figure S2). The central part of the flower possessed spherical pores, which can be ascribed to the interaction between the amine group in TAPP and the sulfonic acid group in PTSA [38].

When 10 μL and 20 μL CNCs were added, due to the added amount not being enough, it has a certain impact on the morphology, but the size has not changed greatly (Figure 4b,c). When 60 μL and 100 μL CNCs were added, the central part of the flowers and petal size had undergone obvious changes. The TEM image of the film with 100 μL CNCs confirmed that the small crystalline of COF-366 was formed in the central part of the flowers (Figure S3). The average size of the petals reached about 0.42 μm and 1.02 μm in the film with 60, 100 μL CNCs, respectively (Figure 4d,e). This experimental phenomenon was similar to the effect of carboxymethyl cellulose (CMC) on calcium carbonate (CaCO_3_) crystals [39]. In the early stages, the morphology of CaCO_3_ crystals changed with the amount of added CMC, and the crystal size increased with the increase in the amount of CMC. In Figure 4b–e, it can be clearly seen that the difference in CNC contents resulted in different morphologies. The reason is probably that the macromolecular framework of CNCs can dynamically respond to external conditions to form various aggregate structures and form a variety of morphologies with counter ions (the monomer of TAPP) through electrostatic interactions [40,41,42]. Adding different amounts of CNCs in the same volume of aqueous solution led to different aggregate structures and then interacts with TAPP to form different forms. With the addition of DHTA, the sulfonic acid groups in CNCs and PTSA were gradually replaced by the aldehyde group, then reacted with the –NH_2_– in TAPP to form imine bonds, and finally the films with different morphologies were obtained. Therefore, the morphology depends on the structure of the CNC aggregate.

Figure 4f showed a single petal morphology of the film with 100 μL CNCs at a higher resolution. There are some holes ranging 0.02 to 0.08 μm on the surface of the petals, which were defects possibly caused by the change in temperatures and the medium components’ concentration during the crystal growth process. In addition, the thickness of the film determined by AFM was approximately 265 nm (Figure S4). Based on the above results, it can be seen that CNCs plays a critical role in the control of single petal size and morphology in COF-366 films.

To further characterize the influence of CNCs on film crystallinity, it is necessary to make the single petal size larger than the TEM beam size as much as possible. The diameter of beam size for HR-TEM is around 350 nm. For SAED, a select area aperture with a physical diameter of 50 μm, corresponding to 700 nm diameter on the image plane, was used. In the COF-366 films with different CNC amounts, only the size of a single petal in the COF-366 film with 100 μL CNCs was larger than the TEM beam, so the following characterizations were mainly focused on the COF-366 film with 100 μL CNCs.

### 3.3. Spectroscopic Characterization of COF-366 Film with 100 μL CNCs

**Figure 5:**
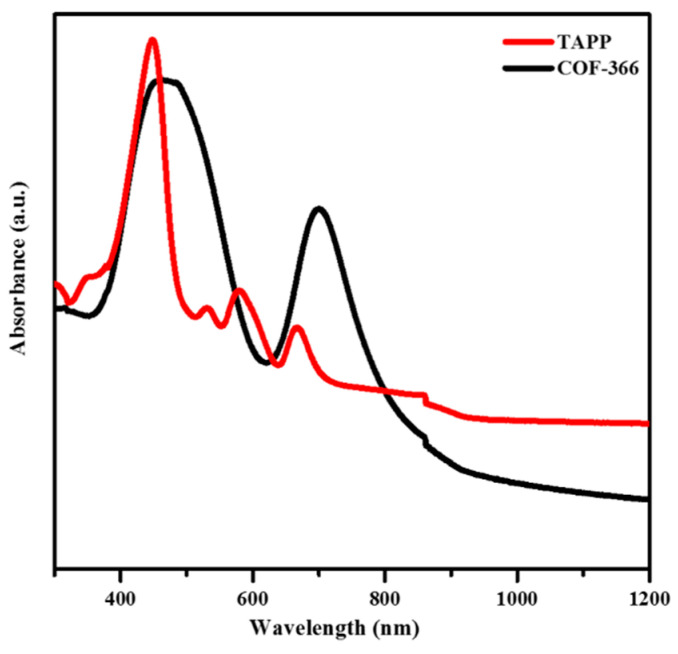
UV–Vis spectra of TAPP and COF-366 film with 100 μL CNCs.

To verify the chemical composition of the COF-366 film, the film was characterized by UV–Vis DRS (Figure 5). UV–Vis spectra (black curve) revealed obvious absorbance of COF-366 film and demonstrated the characteristic Soret (S) band at 471 nm and Q band at 702 nm, which corresponded to porphyrin units. The S-band of COF-366 film was red-shifted by 24 nm compared with TAPP (red curve), which confirmed the existence of extended conjugation in COF-366 film [20].

**Figure 6:**
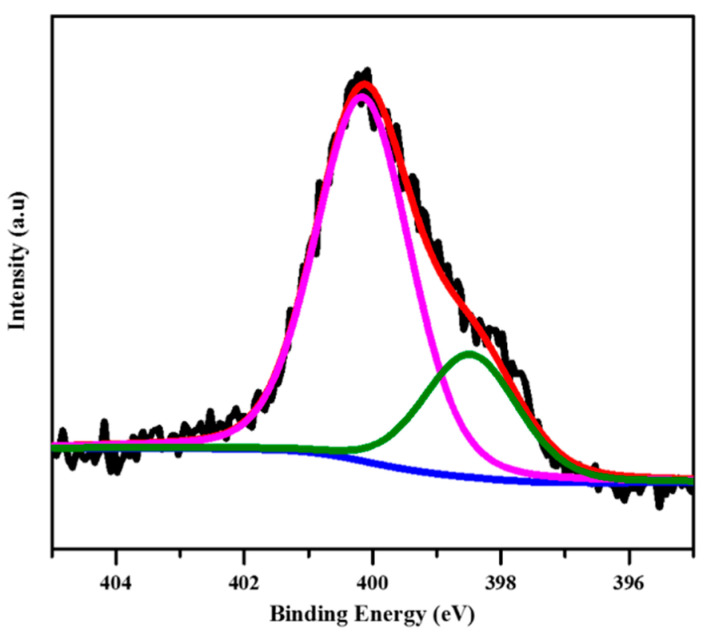
XPS spectra of N1s. There are two peaks with BEs at 400.1 and 398.4 eV, which were attributed to the –NH on the pyrrole nitrogen and the O–H…N=C of the imine nitrogen with hydrogen bonding (purple curve), and the imine nitrogen in the porphyrin unit (green curve), background (blue curve), fitting curve (red curve).

XPS was used to identify the surface composition of the sample by measuring the binding energy of electrons. XPS Peak4.1 software was used to perform curve fitting, and Shirley background was used during the XPS spectra fitting procedure, moreover, all fitted peaks were kept in the shape of the 20% Lorentzian–Gaussian. In the N1s signal of the COF-366 film (Figure 6), the –NH on the pyrrole nitrogen and the O–H…N=C (purple curve) of the imine nitrogen with hydrogen bonding and the imine nitrogen in the porphyrin unit (green curve), corresponded to the peaks of 400.1 and 398.4 eV, respectively. The binding energy intensity ratio between the purple peak and the green peak was 3:1, indicating that each TAPP monomer and DHTA monomer formed four imine bonds in the COF-366 film. There were four binding energy peaks at 284.6, 285.0, 286.1, and 287.2 eV in the C1s signal spectra, belonging to aromatic carbon (C=N, porphyrin units and benzene rings), air pollution, C–N/C–O bonds, and aromatic carbon satellites, respectively (Figure S5). The O1s signal consisted of two peaks with BEs at 533.3 and 531.9 eV, which were attributed to air contamination, and water and hydroxyl groups in the COF-366 film, respectively (Figure S6). These results were consistent with that reported in the literature [15].

### 3.4. TEM and SAED Characterization of COF-366 Films

**Figure 7:**
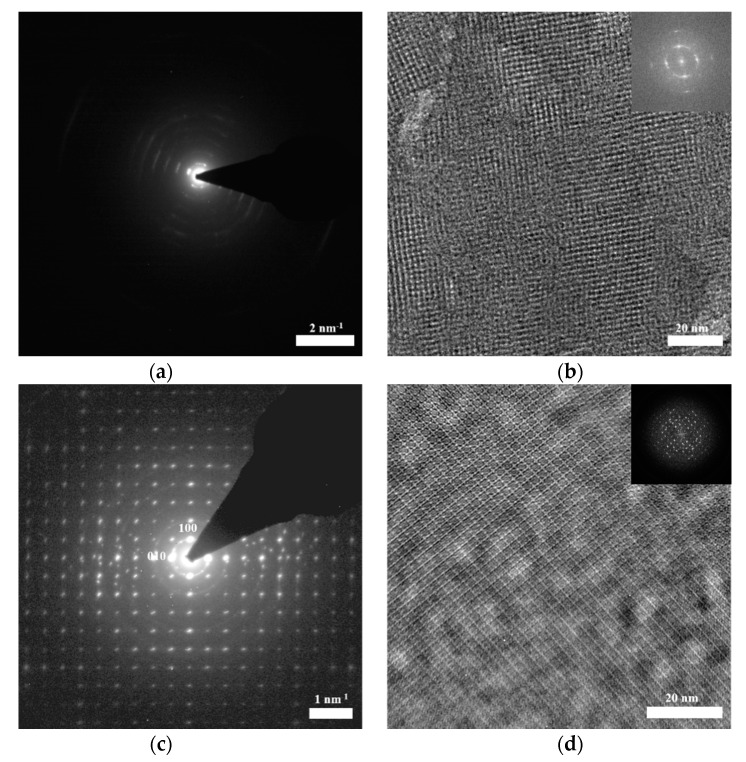

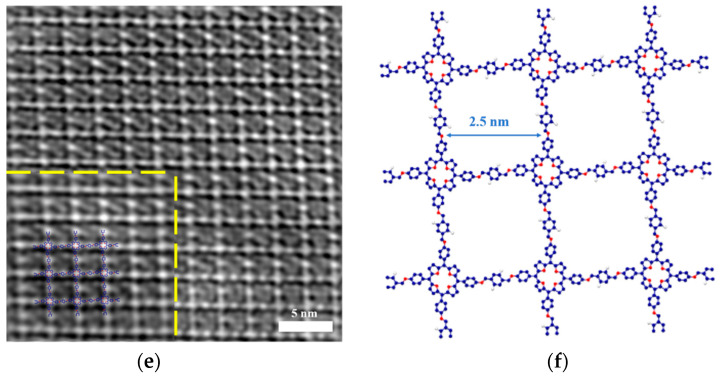
(**a**,**b**) SAED and TEM of COF-366 film without CNCs; (**c**,**d**) SAED and TEM of COF-366 film with 100 μL CNCs; (**e**) HR-TEM image of COF-366 film with 100 μL CNCs (Inset: simulated HR-TEM image with the atomic model overlaid); (**f**) structure simulation of COF-366 by Materials Studio software (carbon: blue, nitrogen: red, oxygen: white).

SAED and TEM measurements were conducted to verify the influence of CNCs on film crystallinity and reveal the structure. SAED pattern showed the nearest reflections occurring at 0.40 nm^−1^ (i.e., 25 Å) (Figure 7a,c). Figure 7a shows the concentric rings, indicating that there are multiple oriented grains in the COF-366 film without CNCs, which was a kind of polycrystalline. Figure 7c exhibited sharp diffraction spots, meaning a high crystallinity of a single petal in the COF-366 film with 100 μL CNCs, which was a single crystal. These results fully demonstrate that the presence of CNCs can effectively improve the COF crystallinity.

The TEM images illustrated the square lattice of COF-366 films (Figure 7b,d). However, compared with the film with 100 μL CNCs, the single-crystalline domains with square symmetry were randomly oriented in the film without CNCs, which suggest the significant influence of CNCs on crystal size.

The HR-TEM image of the film with 100 μL CNCs exhibited a square lattice with 25.0 Å pitch of COF-366 (Figure 7e). Through the image simulation (the inset in Figure 7e), it could be seen that the porphyrin unit appeared bright against a dark background, which was helpful to determine the boundary structure of the domain with the accuracy of the molecule. The parameters and lattice symmetry determined from the HR-TEM images were consistent with the atomic model obtained by Qi et al. [43]. We also optimized the structure simulation of COF-366 using Materials Studio software (Figure 7f), and found that the simulation result had great consistency with our results. Based on the results of the TEM and SAED, it could be seen that CNCs has a significant effect on the crystallinity and crystal size of the film.

## 4. Conclusions

In summary, we successfully synthesized a string of 2D COF films by using CNCs as a template at the air–water interface. The appearances of COF-366 films were flower-like and the size of a single petal in the flowers changed in a certain amount of CNCs. In this work, the sulfate groups (–OSO_3_H) on the CNCs interacted with the protonated amine group (–NH_3_^+^) of TAPP to form CNC–surfactants at the interface. CNC–surfactants with high surface activity assembled at the air–water interface could guide the preorganization of the monomer and the reaction slowly occurred with the addition of DHTA. The existence of CNCs and PTSA slowed the reaction rate, which was conducive to the formation of the crystal film. In addition, the aggregation state of CNCs determined the film morphology. Furthermore, this work found that the presence of CNCs significantly improved the film crystallinity. Based on the effects of CNCs on COFs, we expect that CNCs can be used to assist in the synthesis of large-size crystals of COFs by changing the aggregation forms of CNCs.

## Data Availability

The data presented in this study are available on request from the corresponding author.

## Supplementary Materials

The following are available online at https://www.mdpi.com/article/10.3390/polym13101561/s1, Figure S1: Optical images of COF-366 films. Figure S2: AFM image of the film without CNCs. Figure S3: HR-TEM image of the flower center in COF-366 film with 100 μL CNCs. Figure S4: AFM height image of COF-366 film with 100 μL CNCs. Figure S5: XPS spectra of C1s. Figure S6: XPS spectra of O1s.
