# Supplementary material for: Cellulose Nanocrystals as Template for Improving the Crystallinity of Two-Dimensional Covalent Organic Framework Films
Source: Polymers (Basel). 2021 May 13;13(10):1561. doi: 10.3390/polym13101561 (PMC8152767; doi:10.3390/polym13101561)
Supplement: Supplementary file 1 [file polymers-13-01561-s001.zip › polymers-1181989-supplementary.pdf]

polymers

Supplementary Materials

Cellulose Nanocrystals as Template for Improving the Crystallinity of Two-Dimensional Covalent Organic Framework Films

Yue Li [1], Zhaowei Ou [2], Baokun Liang [3], Jing Yang [4], Ruilian Chen [2], Haoyuan Qi [3,5], Ute Kaiser [3], Wei Hong [2], Xudong Chen [2], Liangwei Du [1,*], Wei Liu [4,*] and Zhikun Zheng [2,*]

1 College of Chemistry and Chemical Engineering, Guangxi University, Nanning 530004, China; LeeYe8@126.com
2 Key Laboratory for Polymeric Composite and Functional Materials of Ministry of Education, School of Chemistry, and State Key Laboratory of Optoelectronic Materials and Technologies, Sun Yat-Sen University, Guangzhou 510275, China; ouzhw6@126.com (Z.O.); chenrlian@mail2.sysu.edu.cn (R.C.); hongwei9@mail.sysu.edu.cn (W.H.); cescxd@mail.sysu.edu.cn (X.C.)
3 Central Facility of Electron Microscopy, Electron Microscopy Group of Materials Science, Universität Ulm, Ulm 89081, Germany; baokun.liang@uni-ulm.de (B.L.); haoyuan.qi@uni-ulm.de (H.Q.); ute.kaiser@uni-ulm.de (U.K.)
4 Key Laboratory of Low-Carbon Chemistry & Energy Conservation of Guangdong Province, Key Laboratory for Polymeric Composite and Functional Materials of Ministry of Education, School of Materials Science and Engineering, Sun Yat-Sen University, Guangzhou 510275, China; yangj329@mail.sysu.edu.cn
5 Center for Advancing Electronics Dresden (cfaed) & Faculty of Chemistry and Food Chemistry, Technische Universität Dresden, Dresden 01062, Germany
* Correspondence: dulily9@163.com (L.D.); liuw226@mail.sysu.edu.cn (W.L.); zhengzhikun@mail.sysu.edu.cn (Z.Z.)

Citation: Li, Y.; Ou, Z.; Liang, B.; Yang, J.; Chen, R.; Qi, H.; Kaiser, U.; Hong, W.; Chen, X.; Du, L.; et al. Cellulose Nanocrystals as Template for Improving the Crystallinity of Two-Dimensional Covalent Organic Framework Films. *Polymers* 2021, *13*, 1561. https://doi.org/10.3390/polym13101561

Received: 29 March 2021
Accepted: 3 May 2021
Published: 13 May 2021

Publisher's Note: MDPI stays neutral with regard to jurisdictional claims in published maps and institutional affiliations.

Copyright: © 2021 by the authors. Licensee MDPI, Basel, Switzerland. This article is an open access article distributed under the terms and conditions of the Creative Commons Attribution (CC BY) license (http://creativecommons.org/licenses/by/4.0/).

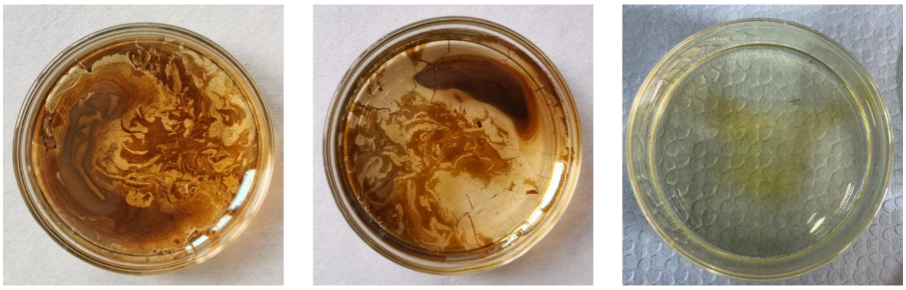

(a) (b) (c)

Figure S1. (**a**) Optical image of COF-366 film with PTSA and CNCs; (**b**) Optical image of COF-366 film without CNCs; (**c**) image of COF-366 film without PTSA.

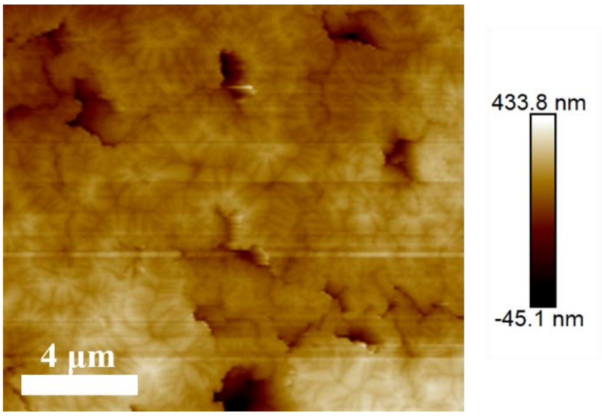

Figure S2. AFM image of the film without CNCs.

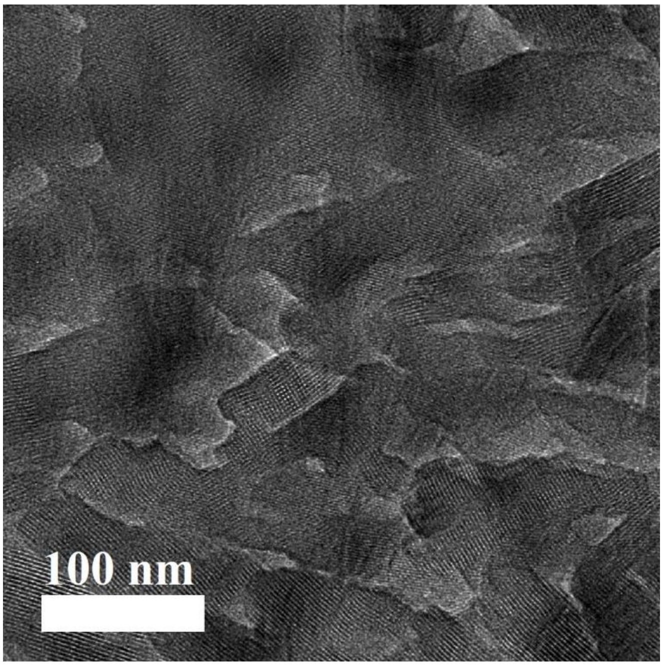

Figure S3. HR-TEM image of the flower center in COF-366 film 100 μL CNCs.

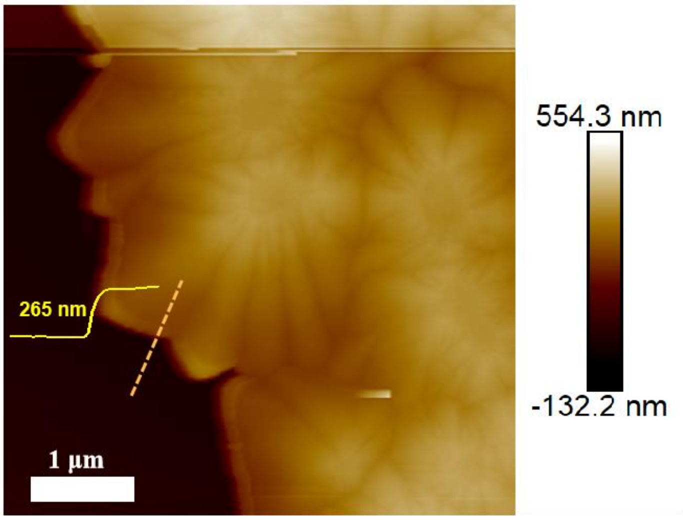

Figure S4. AFM height image of COF-366 film with 100 μL CNCs and the corresponding line profiles.

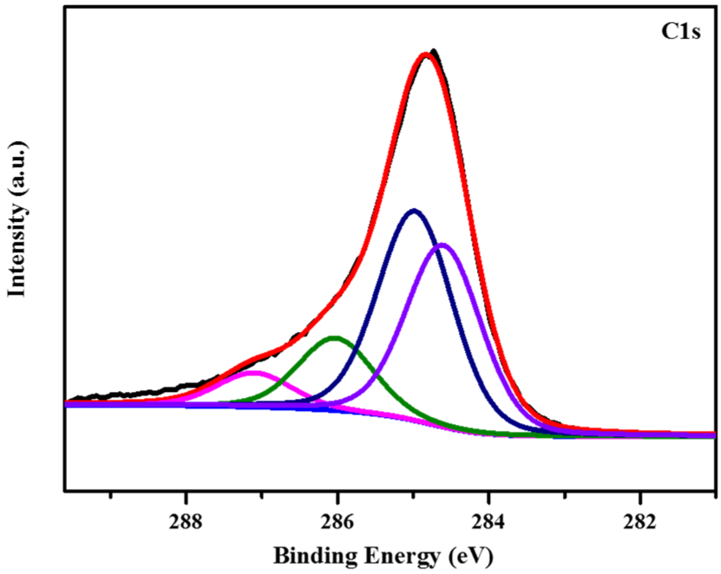

Figure S5. XPS spectra of C1s. There were four binding energy peaks at 284.6, 285.0, 286.1, and 287.2 eV in C1s signal spectra, belonging to aromatic carbon (aromatic: C=N, porphyrin units and benzene rings; pink curve), air pollution (green curve), C-N/ C-O bonds (blue curve), and aromatic carbon satellites (purple curve), respectively; fitting curve (red curve).

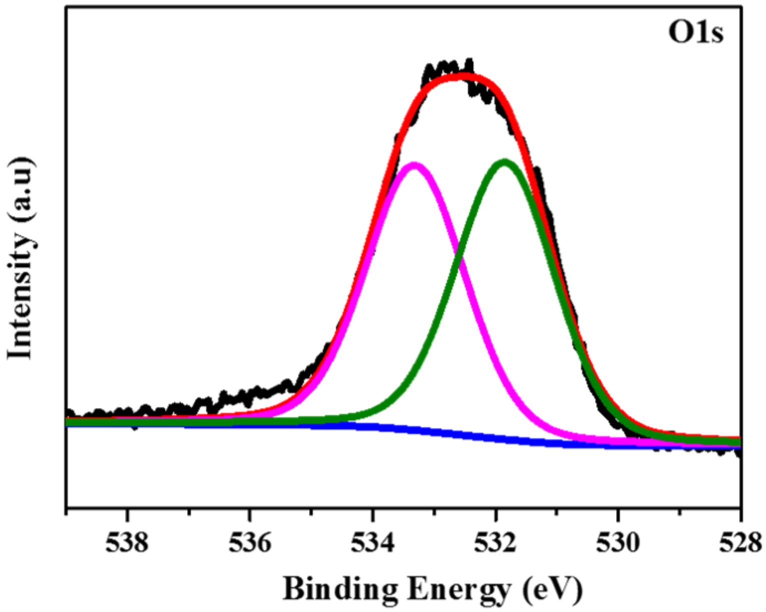

Figure S6. XPS spectra of O1s. There are two peaks with BEs at 533.3 and 531.9 eV, which are attribut-ed to air contamination, and water and hydroxyl groups in the COF-366 film, respectively; background (blue curve); fitting curve (red curve).
